# Individual Variability in Simultaneous Contrast for Color and Brightness: Small Sample Factor Analyses Reveal Separate Induction Processes for Short and Long Flashes

**DOI:** 10.1177/2041669518800507

**Published:** 2018-09-23

**Authors:** Sae Kaneko, Ikuya Murakami, Ichiro Kuriki, David H. Peterzell

**Affiliations:** Research Institute of Electrical Communication, Tohoku University, Miyagi, Japan; Frontier Research Institute for Interdisciplinary Sciences, Tohoku University, Miyagi, Japan; Department of Psychology, The University of Tokyo, Tokyo, Japan; Research Institute of Electrical Communication, Tohoku University, Miyagi, Japan; College of Psychology, John F. Kennedy University, Pleasant Hill, CA, USA; School of Optometry - Vision Sciences, University of California, Berkeley, CA, USA

**Keywords:** color, individual differences, lightness, brightness, color appearance, chromatic induction, simultaneous contrast

## Abstract

In classic simultaneous color contrast and simultaneous brightness contrast, the color or brightness of a stimulus appears to shift toward the complementary (opposite) color or brightness of its surrounding region. Kaneko and colleagues proposed that simultaneous contrast involves separate “fast” and “slow” mechanisms, with stronger induction effects for fast than slow. Support for the model came from a diverse series of experiments showing that induction by surrounds varying in luminance or color was stronger for brief than long presentation times (10–40 vs. 80–640 ms). Here, to further examine possible underlying processes, we reanalyzed 12 separate small data sets from these studies using correlational and factor analytic techniques. For each analysis, a principal component analysis of induction strength revealed two factors, with one Varimax-rotated factor accounting for brief and one for long durations. In simultaneous brightness experiments, separate factor pairs were obtained for luminance increments and decrements. Despite being based on small sample sizes, the two-factor consistency among 12 analyses would not be expected by chance. The results are consistent with separate fast and slow processes mediating simultaneous contrast for brief and long flashes.

## Introduction

Simultaneous color contrast and simultaneous brightness^1^ contrast are two classic phenomena in which spatial context affects perception. In simultaneous color contrast, a patch of color in a visual scene induces a shift in the color appearance of other nearby stimuli that appear simultaneously. A highly saturated red annulus, for instance, will induce a greenish hue into the neutral gray central test stimulus which it surrounds (e.g., Kinney, 1965). Similarly, it will cause a central test stimulus that appears unique yellow in isolation to appear greenish yellow (e.g., Akita, Graham, & Hsia, 1964). In simultaneous brightness contrast, a pattern in a visual scene induces a shift in the brightness of other stimuli. A high-luminance white annulus, for instance, will cause the medium gray central test stimulus which it surrounds to appear darker or black (e.g., Cicerone, Volbrecht, Donnelly, & Werner, 1986; Heinemann, 1955; Shinomori, Shefrin, & Werner, 1997; Volbrecht, Werner, & Cicerone, 1990).

A recent discovery is that simultaneous contrast induction is typically much stronger for brief than long presentation durations (Kaneko & Murakami, 2012; Robinson & de Sa, 2008). The strongest induction effects are seen in the briefest flashes and decline exponentially with increasing duration. That the induction effect is stronger for brief than long flashes was first reported for simultaneous brightness contrast (Robinson & de Sa, 2008; see also Blakeslee & McCourt, 2008). Kaneko and colleagues (Kaneko, Anstis, & Kuriki, 2017; Kaneko & Murakami, 2012) further confirmed these findings and used various equiluminant chromatic surrounds to show that simultaneous color contrast was also stronger for shorter durations. These studies suggest the fast-responding mechanisms are underlying simultaneous contrast. In contrast to these recent discoveries, others have demonstrated that brightness induction was only seen with a slow-modulating stimulus, which suggests that sluggish mechanisms are underlying simultaneous contrast (De Valois, Webster, De Valois, & Lingelbach, 1986; Gunther & Dobkins, 2005; Rossi & Paradiso, 1996).

These findings have led Kaneko and colleagues to propose that the processes underlying simultaneous contrast effects involve separate fast and slow mechanisms, with fast mechanisms typically showing stronger induction effects than slow mechanisms (Kaneko et al., 2017; Kaneko & Murakami, 2012). The hypothesized fast processes are responsible for induction effects observed for short (10–40 ms) stimulus durations. The slow processes are hypothesized to mediate the effects >80 ms after stimulus onset, that is, for longer presentation durations. We note that the fast versus slow induction processes proposed by Kaneko and colleagues resemble widely studied transient versus sustained channels in vision (Kulikowski & Tolhurst, 1973), but we believe that they are different than these classic processes. We discuss this point further in Brightness Experiment 1.

The purpose of the present study is to test the fast versus slow process hypothesis using a methodological alternative to analyzing individual and group averages. Previously, typical analyses examine the shapes of curves across experimental conditions, assessing significant differences among these conditions (e.g., across stimulus durations).

However, we have reanalyzed the data of Kaneko and Murakami (2012) and Kaneko et al. (2017) by examining statistical factors underlying individual differences in the data (e.g., Mollon, Bosten, Peterzell, & Webster, 2017; Peterzell, 2016; Peterzell, Werner, & Kaplan, 1991). The general premise of the individual differences or factor analytic approach is that the measured variability among observers can be informative about the underlying visual mechanisms. The rationale is as follows. If the data under Condition A and Condition B are driven by mechanism no. 1, and data under Conditions C and D are driven by mechanism no. 2, then individual data points for Conditions A and B should be highly correlated, and data points for Conditions C and D should be highly correlated, whereas individual data points for Conditions A and C should not be correlated as much. Factor analysis is a statistical technique used to examine these correlational structures in the sets of individual data points and reveal a few factors to best explain the variation in the observed data. The number of extracted factors and their factor loadings should reflect the number of underlying mechanisms and how they contribute to data observed under different conditions. See Peterzell (2016) and Mollon et al. (2017) for more details.

The factor analytic approach has been used in previous studies to elucidate temporal processes in data (Billock & Harding, 1996; Dobkins, Gunther, & Peterzell, 2000; Peterzell, 1993; Peterzell, Dougherty, & Mayer, 1997; Strasburger, Murray, & Remky, 1993), and chromatic processes in data (Burt, 1940, 1946; Dobkins et al., 2000; Emery, Volbrecht, Peterzell, & Webster, 2017a, 2017b, 2017c; Gunther & Dobkins, 2003; Hamer, et al., 2016; Jones & Jones, 1950; MacLeod & Webster, 1988; Mayer, Dougherty & Hu, 1995; Peterzell, 2011; Peterzell, Chang, & Teller, 2000; Peterzell, Chang, Kelly, Hartzler & Teller, 1997; Peterzell et al., 1996, 2016; Peterzell & Teller, 2000; Pickford, 1951; MacLeod, & Webster, 1988). (See also Bosten & Mollon, 2010).

We applied factor analysis to 12 different subsets of data collected by Kaneko and colleagues (Kaneko et al., 2017; Kaneko & Murakami, 2012). These 12 data subsets, along with the four experiments from which they were taken, are summarized in Table 1 and described throughout this article. In particular, we applied factor analysis to data subsets from three different experiments originally collected by Kaneko and Murakami (2012), and one experiment collected by Kaneko et al. (2017). The first two experiments examine simultaneous brightness contrast (data sets 1–7), whereas the last two examine simultaneous color contrast (data sets 8–12). Because we only included experiments with *n* ≥ 5 observers, some other data from the two studies were not reanalyzed.

**Table 1.** Summary of Data Sets Reanalyzed, Factor Analyses Performed, and Results.

Name of data set	Source of data set	Design	Separate factor analyses	Result
Brightness Experiment 1	Kaneko and Murakmi (2012), Experiment 1, *n* = 7	Test stimulus: gray. Inducers: two luminance increments (white, light gray) and two luminance decrements (dark gray, black). Gap conditions: no gap, narrow gap, wide gap. Durations: 10 ms, 500 ms.	1) 0°: no gap 2) 0.25°: narrow gap 3) 1°: wide gap	1) four factors: *fast* and *slow* for increments; *fast* and *slow* for decrements 2) four factors: *fast* and *slow* for increments; *fast* and *slow* for decrements 3) four factors: *fast* and *slow* for increments; *fast* and *slow* for decrements
Brightness Experiment 2	Kaneko and Murakmi (2012), Experiment 5, *n* = 6	Test stimulus: gray. Inducers: two luminance increments (white, light gray) and two luminance decrements (dark gray, black). Durations: 10 to 640 ms.	4) white (increment) 5) light gray (increment) 6) dark gray (decrement) 7) black (decrement)	4) two factors: *fast* and *slow* 5) two factors: *fast* and *slow* 6) two factors: *fast* and *slow* 7) two factors: *fast* and *slow*
Color Experiment 1	Kaneko and Murakmi (2012), Experiment 4, *n* = 6	Test stimulus: gray. Inducers: purple, lime, red, green. Durations: 10 to 640 ms.	8) purple 9) lime 10) red 11) green	8) two factors: *fast* and *slow* 9) two factors: *fast* and *slow* 10) two factors: *fast* and *slow* 11) two factors: *fast* and *slow*
Color Experiment 2	Kaneko, Anstis, and Kuriki (2017), Experiment 2, *n* = 5	Test stimulus: unique yellow. Inducers: green, greenish yellow, reddish yellow, red. Durations: 10 ms, 500 ms.	12) All conditions (Four Inducers × Two Durations)	12) two factors: *fast* and *slow*

For each subset of data, we asked if fast and slow factors were evident, and if the two factors were necessary and sufficient to account for systematic variability among individuals’ data. We predicted, following Kaneko and Murakami (2012), that individual variability in data would reveal factors consistent with fast and slow processes operating over different duration ranges (short and long, respectively). Our consistent finding of systematically tuned fast and slow factors across all 12 data sets would be highly unlikely due to chance alone.

## Brightness Experiment 1

The first three sets of data that we reanalyzed were taken from a single experiment reported by Kaneko and Murakami (2012, Experiment 1 of 5) and listed as Brightness Experiment 1 in Table 1.

### Overview

In this experiment, seven observers viewed a test stimulus that was a 1° diameter disk whose color was uniform gray at 33 cd/m^2^. On different trials, the central test was presented for one of two durations (either 10 or 500 ms), within one of four brightness inducing surrounds (0–66 cd/m^2^), with one of three concentric spatial gaps separating test and inducer (0–1°). On each trial, observers adjusted the luminance of a comparison stimulus to be a perceptual match to the central gray test. For each trial’s perceptual match, the percentage deviation in luminance of the comparison stimulus from the gray test stimulus was recorded as a measure of “induction strength.” Thus, “induction strength” refers in this experiment to the strength of the simultaneous brightness contrast that was recorded for each condition. In all analyses, the absolute value of this induction strength estimate was used.

### Methods

Essential details of the experiment are provided here. For full details of each study, refer to Kaneko and Murakami (2012).

#### Observers

Seven observers with normal or corrected-to-normal visual acuity participated, including one of the authors (S. K.). Their ages were between 18 and 28 years.

#### Apparatus and stimuli

**Figure 1.**
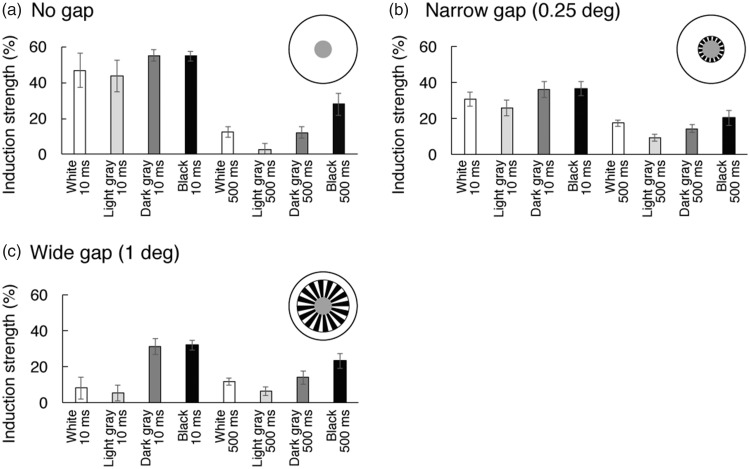
Induction strength observed in Brightness Experiment 1 (*n* = 7). Mean ± *SE*. Panels (a) to (c) show data for the no gap condition (a), narrow gap condition (b), and wide gap condition (c), respectively. Adapted from Kaneko and Murakami (2012).

Stimuli were presented on a CRT display (Mitsubishi Diamondtron M2, 22″, refresh rate 100 Hz) under computer control. All stimuli were generated using the MATLAB programming environment with Psychtoolbox (Brainard, 1997; Pelli, 1997). The test stimulus was a 1° diameter disk whose color was uniform gray at 33 cd/m^2^. Another disk of the same size was presented as a comparison stimulus, whose luminance setting was adjustable. The test was embedded at the center of a larger uniform disk, the inducer. The outer diameter of the inducer was either 16.5° (brightness experiment) or 10° (color experiment). The inner diameter of the inducer was equivalent to the diameter of the test stimulus. In Brightness Experiment 1, one of three “gaps” was inserted between the test and the inducer. The gap was a patterned ring with 32 windmill-like sectors, painted either white or black (see insets of Figure 1). Gap width conditions were 0° (“no gap”), 0.25° (“narrow gap”), or 1° (“wide gap”). Brightness inducer conditions were “white” (66 cd/m^2^), “light gray” (53 cd/m^2^), “dark gray” (13 cd/m^2^), and “black” (0 cd/m^2^). The test stimulus with the inducer and the comparison stimulus were presented side-by-side on the display (center-to-center distance between the two stimuli was 16.5°, and the rest of the display was filled with a black-and-white random dot (half of the dots were black) noise pattern. The noise pattern, whose mean luminance was equal to the luminance of the test stimulus, was used in order to prevent the area from affecting the brightness of the stimuli (the inducer, the comparison, and the test stimulus). The duration of the test plus the inducer was either 10 or 500 ms in Brightness Experiment 1, and these duration conditions were run in separate sessions.

#### Procedure

Observers were instructed to compare the brightness of the test stimulus with that of the comparison stimulus and to adjust the comparison stimulus so that the two stimuli appeared equally bright. The observer adjusted the luminance level of the comparison stimulus by keypress. The inducer and the test stimuli were presented repeatedly with 500-ms blank intervals (where the inducer and the central test stimulus were replaced by a uniform mean-gray field) until the observer made a satisfactory match. The adjustable comparison stimulus was always visible. Each observer made 12 matches per condition.

The experimental procedure was approved by the review board of The University of Tokyo, where all the experiments were performed. All the observers gave their written informed consent.

### Results: Group Averages (Kaneko & Murakami, 2012)

From these data, Kaneko and Murakami reported that simultaneous brightness contrast using a gray central test was stronger for short (10 ms) than long durations (500 ms), consistent with their later conclusion that brightness induction drops exponentially with longer stimulus durations. They showed the effect for the four surround conditions, including two luminance increments (white, light gray) and two decrements (dark gray, black) relative to the gray central test stimulus.

In addition to studying the effects of stimulus duration upon the strength of simultaneous contrast, Brightness Experiment 1 further examined spatiotemporal properties of simultaneous brightness contrast. Thus, Kaneko and Murakami included three gap conditions, including concentric spatial gaps separating the test and inducer of 1.0° (wide gap), 0.25° (narrow gap), and 0° (no gap). The induction seen for a short (10 ms) duration flash was greatly reduced when a concentric spatial gap was introduced between the 1° test disk and the inducer annulus. However, at long (500 ms) durations, the simultaneous brightness contrast effect was unaffected by concentric spatial gaps up to 1°.

The results supporting these conclusions are shown in Figure 1. Mean induction strength for the seven observers is shown. Figure 1(a) shows that for all inducer conditions, the 10-ms duration yielded much stronger induced brightness than the 500-ms duration when there was no spatial gap between the test and the inducer. However, Figure 1(b) and (c) shows that the effect of the spatial gap on induction strength varied depending on the stimulus duration. For the 10-ms duration, the gap significantly reduced the induction strength regardless of the inducer condition. On the other hand, the gap did not affect the induction strength when the stimulus duration was 500 ms.

### Results: Factor Analysis of Kaneko and Murakami (2012) Data

#### Predictions

We analyzed Kaneko and Murakami (2012)’s data obtained for the three gap conditions separately, in three separate analyses. In the three data sets analyzed here (wide, narrow, and no gap conditions), like all data analyzed in the current article, we predicted in each case that individual variability in data would reveal factors consistent with fast and slow processes operating over different duration ranges (short and long, respectively). Although the concentric gaps weaken simultaneous brightness contrast for short durations, this does not imply that the fast mechanisms disappear with wide gaps.

Moreover, there was reason to predict that separate sets of factors would be obtained for increments and decrements. There is a considerable psychophysical and neurophysiological literature suggesting that increments and decrements are processed by separate mechanisms, specifically the “On” and “Off” pathways of the mammalian visual system that originate at the retina (Fiorentini, Baumgartner, Magnussen, Schiller, & Thomas, 1990; Jung, 1964; Schiller, 1992). Differences in induction effects for increments and decrements have been shown for brightness contrast, and these may reflect separate underlying mechanisms (Kingdom, 2003; Hong & Shevell, 2004).

#### Results and discussion

**Figure 2.**
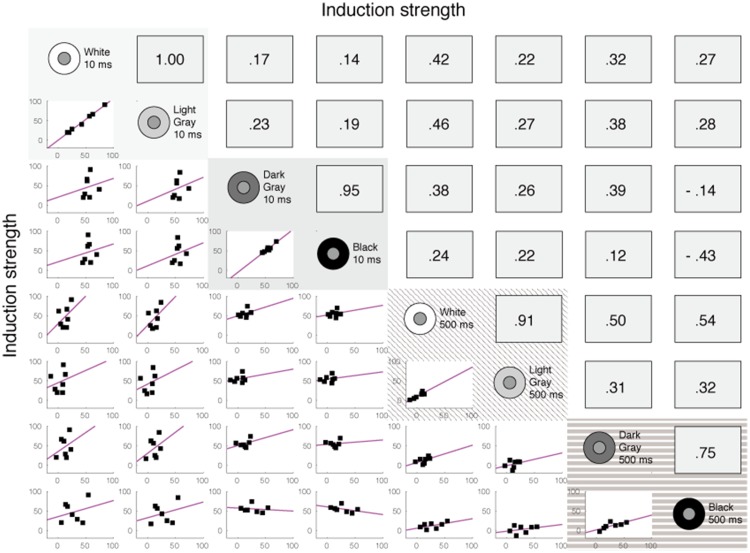
Scatterplot and correlation matrices for Brightness Experiment 1 data, no gap condition. See text for details.

Even without conducting a statistical factor analysis, one can see the pattern of correlations among the conditions by viewing all the data simultaneously and thus conducting an “intuitive factor analysis” (Peterzell, 2016). This is accomplished by viewing Figure 2, a correlation matrix and scatterplot matrix for all combinations of conditions in the “no gap” condition. The lower left half (i.e., below the diagonal) of the overall matrix shows scatterplot matrices, while the top right half (i.e., above the diagonal) of the matrix shows the correlation coefficients (Pearson’s *r*) for the corresponding pair of conditions. For instance, the first scatterplot in the first column (second row) of Figure 2 shows simultaneous contrast induction strengths obtained for *n* = 7 observers when viewing the light gray, 10-ms condition, plotted as a function of the induction strengths obtained for the same *n* = 7 observers when viewing the white, 10-ms condition. A magenta line shows the best-fitting regression line. The Pearson correlation corresponding to this first scatterplot is shown in the second column (first row), *r*(1, 5) = 1.00.

Similarly, the bottom scatterplot in the sixth column of Figure 2 shows simultaneous contrast induction strengths obtained for *n* = 7 observers when viewing the black, 500-ms condition, plotted as a function of the induction strengths obtained for the same *n* = 7 observers when viewing the light gray, 500-ms condition. Again, a magenta line shows the best-fitting regression line. The Pearson correlation corresponding to this scatterplot is shown in the last column (sixth row), *r*(1, 5) = 0.32.

**Figure 3.**
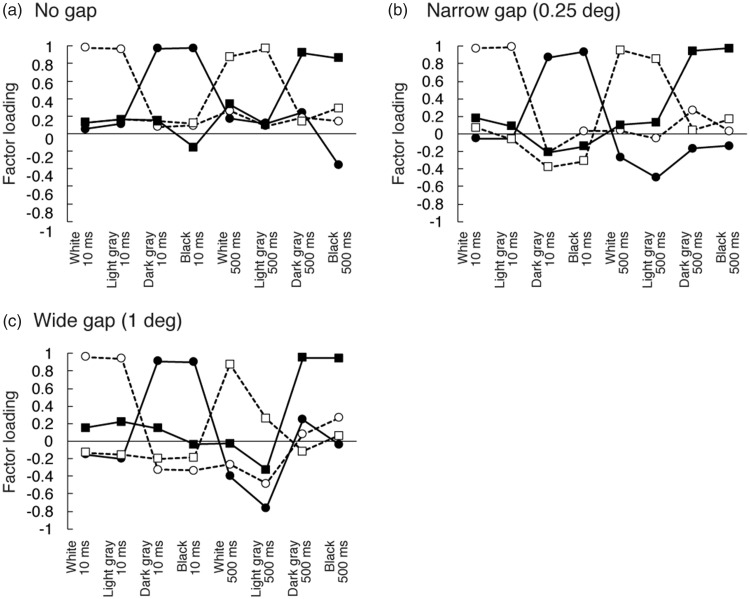
Factor loading patterns obtained for three factor analyses from Brightness Experiment 1. Each panel shows the four-factor solution for data from (a) the no gap condition, (b) the narrow gap condition, or (c) the wide gap condition. Lines with different symbols represent the four factors. Each factor from each of the three data sets has high (>.4) loadings only for two conditions, except for one factor (open square with a dashed line) in wide gap condition which has high loading value for only one condition.

By viewing Figure 2 in this way, four visible factors (i.e., regions of comparatively high correlation) are evident, as denoted by four shaded rectangular boxes, with separate boxes for fast increments (white and light gray inducers, 10-ms conditions), fast decrements (dark gray and black inducers, 10-ms conditions), slow increments (white and light gray inducers, 500-ms conditions), and slow decrements (white and light gray inducers, 500 ms conditions). These four visible factors illustrate how the raw data embody the factors computed in the next step of the analysis and shown in Figure 3(a). The patterns of scatterplot and correlational matrices obtained for the narrow gap condition and wide gap conditions (not shown) were highly similar to those shown here for the no gap condition.

Thus, by eyeballing the correlational patterns, we can “see” or intuit the underlying factors by finding clusters with highly correlated data combinations. In this case, four factors clearly emerged which were highlighted as differently shaded areas in Figure 2. Each factor is a “doublet,” in that it is comprised of two and only two conditions.

The factor analytic approach to estimating the number and nature of visual mechanisms has been described in detail elsewhere (e.g., Emery et al., 2017a, 2017b; Peterzell, 2016; Peterzell et al., 1991, 2000; Peterzell & Teller, 1996). Here we only briefly describe the steps of the analysis. First, we used principal component analysis (PCA) to extract components, or factors, from the correlation matrix of the data. Then, the components were orthogonally rotated using the Varimax criterion to approximate Thurstonian “simple structure,” which maximizes the number of zero or near zero loadings. To be deemed significant, factors needed to rise above the “scree” in visual scree tests, and the rotated factor loadings for each factor needed to exceed 0.4 for at least two adjacent variables (following Gorsuch, 1983; MacLeod & Webster, 1988. An exception was made for third factor, a “singlet” factor in the wide gap condition). In these analyses, “adjacent” refers to variables that are close along a dimension (i.e., white and light gray are similar, whereas white and dark gray are more dissimilar; 10 and 20 ms are similar, whereas 10 and 160 ms are dissimilar, or nonadjacent). Finally, factor loadings computed from these analyses were inspected and compared with predictions. Each of these factor loadings, like *any* factor loading, provides the correlation between an input variable (i.e., the data for one Duration Condition × Inducer Condition) and a factor (i.e., one of the factors obtained from the Varimax-rotated PCA).

We predicted, as noted earlier, the emergence of fast and slow factors. That is, we predicted that separate factors would emerge for short and long durations, with stimuli of short and long durations loading onto separate factors. A randomly varying data set would not be expected to yield factors tuned systematically to short and long temporal conditions. The existence of such factors in our data would therefore support our hypothesis.

Figure 3 shows the factor loadings for the four-factor solution, computed independently for each of the three gap conditions. The results of each of three analyses are consistent with what was estimated from the scatterplot matrix (Figure 2). In each of three analyses, each of the four factors has high factor loading values for only two conditions and has low factor loadings for the other six conditions. These four factors accounted for 98.5% (no gap condition), 99.5% (narrow gap condition), and 98.6% (wide gap condition) of the total variance. We call these factors “increment/fast” (high loadings for white, light gray, 10 ms), “decrement/fast” (high loadings for dark gray, black, 10 ms), “increment/slow” (high loadings for white, light gray, 500 ms), and “decrement/slow” (high loadings for dark gray, black, 500 ms), respectively. For luminance increments, there are clearly separate factors for brief and long durations. For luminance decrements, there are clearly separate factors for brief and long durations. The two factors obtained for increments are clearly separate from the two factors obtained for decrements.

Most remarkably, note that these four factors and their factor loading patterns were nearly identical for all three gap conditions. The consistency across conditions is clear despite the significant differences in induction strength shown in Figure 1. The similarity of the four factors across the three conditions highlights the reliability of the results.

Here, we discuss the relationship between fast or slow processes suggested here and classic transient or sustained channels (Kulikowski & Tolhurst, 1973). We claim that fast process is separate from transient channel because of their spatial properties. The classic transient channel is tuned to high temporal frequencies (i.e., motion) and low spatial frequencies (Anderson & Burr, 1985; Kulikowski & Tolhurst, 1973). On the other hand, the fast process in simultaneous brightness contrast we discuss in the current analysis is greatly affected by spatial gaps (Figure 1), which suggests that the fast process has a small “receptive field.” While the two are not mutually exclusive concepts, we now tentatively suggest that the fast process is not the transient channel and slow process is not the sustained channel.

## Brightness Experiment 2

The next four data sets we reanalyzed were taken from a single experiment reported by Kaneko and Murakami (2012, Experiment 5 of 5) and listed as Brightness Experiment 2 in Table 1.

### Overview

This experiment of Kaneko and Murakami was designed to replicate the simultaneous brightness contrast effects reported in the first simultaneous brightness experiment (i.e., Experiment 1 of 5 from their article, and Brightness Experiment 1 earlier) for the four inducers (white, light gray, dark gray, and black), while extending the findings to seven stimulus durations ranging from 10 to 640 ms. No gaps between the center test and surrounds were used in this experiment. For all inducer conditions, simultaneous contrast induction peaked at 10 ms (the shortest duration studied) and declined rapidly with increasing duration.

### Methods

Essential details of the experiment are provided here. For full details of each study, refer to Kaneko and Murakami (2012).

#### Observers

Six observers with normal or corrected-to-normal visual acuity participated, including one of the authors (S. K.). Their ages were between 18 and 28 years.

#### Apparatus, stimuli, and procedure

The apparatus, stimuli, and procedure were identical to Brightness Experiment 1, except for the following*.* On any trial, presentation duration was one of the following: 10, 20, 40, 80, 160, 320, or 640 ms. These duration conditions were presented in a pseudorandom order within a session. All stimuli contained no gaps between the central gray test and inducing surround. Each observer made 24 matches per condition.

### Results: Group Averages (Kaneko & Murakami, 2012)

**Figure 4.**
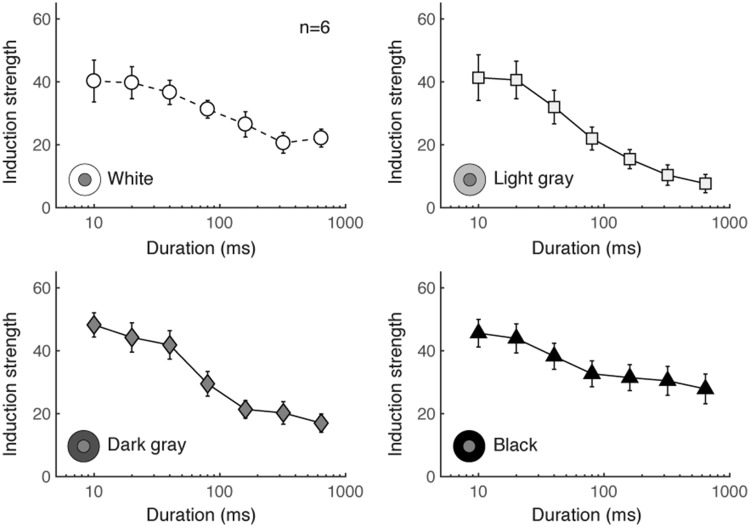
Mean induction strength for all four inducer conditions, for Brightness Experiment 2. Error bars indicate standard error. Adapted from Kaneko and Murakami (2012).

Mean induction strength for each inducer condition is shown in Figure 4. For all conditions, the induction strength was strongest at the shortest (10 ms) duration and exponentially decayed with increasing duration (note that the *x* axis is logarithmic).

### Results: Factor Analysis of Kaneko and Murakami (2012) Data

#### Predictions

Again, we predicted, following Kaneko and Murakami (2012), that individual variability in the data would reveal factors consistent with fast and slow processes operating over different duration ranges (short and long, respectively). The use of seven stimulus durations rather than just two (as in Brightness Experiment 1) was predicted to provide a clear estimate of the stimulus durations influenced by fast and slow processes. The fast and slow processes were predicted to emerge reliably across the four inducer conditions.

#### Results and discussion

For data from this experiment, we factor analyzed each of the four inducer conditions separately, in four different data sets. For these analyses, we found two-factor solutions to be optimal in each instance, with strong evidence of systematic tuning following Varimax rotation. For each inducer condition, the two factors accounted for 96.3% (white), 93.4% (light gray), 92.8% (dark gray), and 95.9% (black) of the total variance.

**Figure 5.**
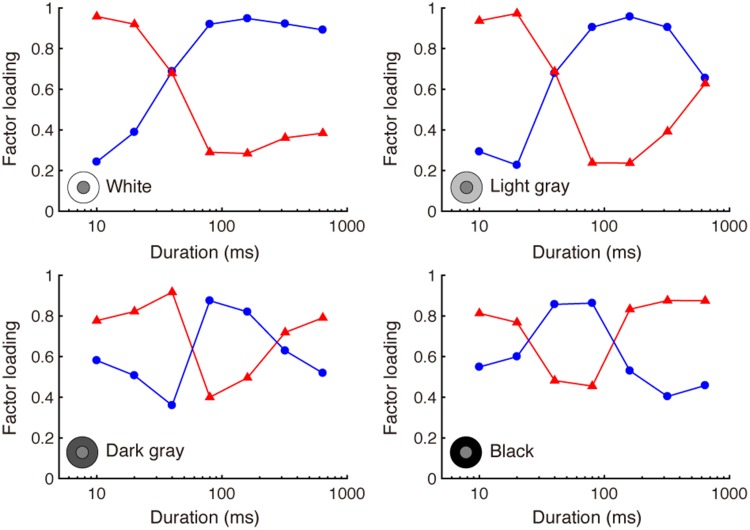
Factor loading patterns for each inducer condition, for Brightness Experiment 2.

Factor loading values are shown in Figure 5. For increments (white and light gray), there was a clear dissociation between one factor whose loading is high only for short (≤40 ms) durations to another factor whose loading is high only for long (>40 ms) durations. We named the former fast and the latter slow, respectively, consistent with the theory of Kaneko and Murakami (2012).

A similar dissociation was found for decrement inducers, but it was less clear. The fast factor that had high loadings at short durations also had high loadings for long durations, while the other slow factor had high loadings for mid-range duration conditions. These less clear results could reflect random effects due to the small sample size, but the systematic tuning of the factor loadings suggests an effect that may be real. The results for those stimulus conditions could mean that the fast induction mechanism turns on quickly and strongly but decays in strength fairly slowly, while the slow induction mechanism turns on late but turns off early. We think this could, for instance, explain the performance for the black inducer in Figure 5. The idea is that the fast mechanism continues to operate, decaying only slightly in strength, but the slow mechanism, when it is on, dominates the response and individual variability in the response. This would cause loadings for the fast factor to dip while the slow factor is operating. That does not necessarily go against the idea of a fast and a slow mechanism, but it does suggest that the slow mechanism may be somewhat transient under certain stimulus conditions. A larger sample size will be required to further investigate and resolve this issue.

## Color Experiment 1

The next four data sets we reanalyzed were taken from a single experiment reported by Kaneko and Murakami (2012, Experiment 4 of 5) and listed as Color Experiment 1 in Table 1.

### Overview

This experiment was comparable to the Brightness Experiment 2, but inducers were varied in hue instead of luminance. All the colors used in this experiment were on an equiluminant plane in Derrington-Krauskopf-Lennie color space (Derrington, Krauskopf, & Lennie, 1984). In this experiment, observers adjusted the *saturation* of the comparison stimulus (with its hue fixed) to match with the apparent color of the test stimulus. Since the color coordinates of the inducer and test stimulus were fixed, a higher saturation of the comparison stimulus at the perceptual match meant stronger simultaneous color contrast. The percentage deviation in distance between the perceptually matched comparison stimulus and the origin was taken as the induction strength.

This experiment examined the effects of various stimulus durations upon simultaneous color contrast. Inducers were of four colors (red, green, purple, and lime) that were equiluminant to the gray test stimulus (heterochromatic flicker photometry was performed for each observer to ensure equiluminance of the colors). Seven stimulus durations were used, ranging from 10 to 640 ms. For all inducer conditions, simultaneous contrast peaked at 10 ms (the shortest duration studied) and declined rapidly with increasing duration.

### Methods

#### Observers

Six observers with normal or corrected-to-normal visual acuity participated, including one of the authors (S. K.). Their ages were between 18 and 28 years.

#### Apparatus, stimuli, and procedure

The apparatus, stimuli, and procedure were identical to Brightness Experiment 2, except for the following. Colors of all inducers were on the equiluminant plane passing through the center in the Derrington-Krauskopf-Lennie color space (Derrington et al., 1984). Four colors were used for inducer colors, including two along the cardinal L–M axis (red and green), and two along the cardinal S − (L + M) axis (purple and lime). Inducers’ coordinates are [0.11, 0.00] (red), [−0.11, 0.00] (green), [0.00, 0.67] (purple), and [0.00, −0.67] (lime), where the first value indicates the color’s distance from the origin along the cardinal L–M axis, and the second indicates the distance along the cardinal S − (L + M) axis.

The outer diameter of the inducer was 10°. Another disk of the same size as the central gray test (1° diameter) was presented as a comparison stimulus, whose saturation was adjustable. The test stimulus with the inducer and the comparison stimulus were presented side-by-side on the display (center-to-center distance between the two stimuli was 5°). Observers were instructed to compare the color of the test stimulus with that of the comparison stimulus and to adjust the saturation of the comparison to match the appearance of the test. The initial saturation of the comparison in each trial was randomly chosen from the adjustable range. Once perceptual matches were set, induction strength measures were recorded. Induction strength was defined as the percentage deviation in distance from the origin to the perceptually matched comparison stimulus along the inducer’s axis (e.g., L−M axis when the inducer was red).

### Results: Group Averages (Kaneko & Murakami, 2012)

**Figure 6.**
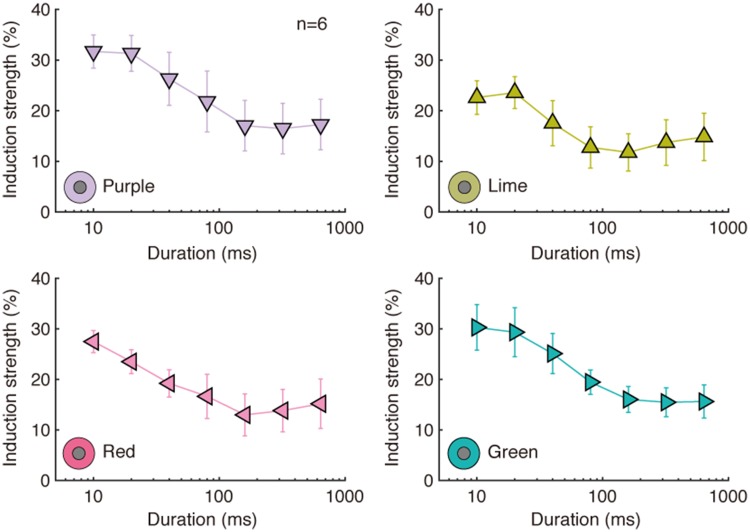
Mean induction strength for all color inducer conditions (Color Experiment 1). Mean ± *SE*. Induction strength was defined as the percentage deviation in distance from the origin to the perceptually matched comparison stimulus. A positive value indicates that the apparent color of the test shifted in the complementary direction of the inducer. Adapted from Kaneko and Murakami (2012).

The mean induction strength for four color inducer conditions is shown in Figure 6. In this experiment, Kaneko and Murakami reported that simultaneous color contrast exhibited the same pattern as simultaneous brightness contrast across seven durations ranging from 10 to 640 ms. Stronger induction effects were observed for short compared with long durations. For each of the four conditions, chromatic induction peaked at 10 ms (the shortest duration studied) and declined rapidly with increasing duration.

### Results: Factor Analysis of Kaneko and Murakami (2012) Data

#### Predictions

Again, we predicted, following Kaneko and Murakami (2012), that individual variability in data would reveal factors consistent with fast and slow processes operating over different duration ranges (short and long, respectively). The fast and slow processes were predicted to emerge reliably for the four chromatic surround conditions.

#### Results and discussion

**Figure 7.**
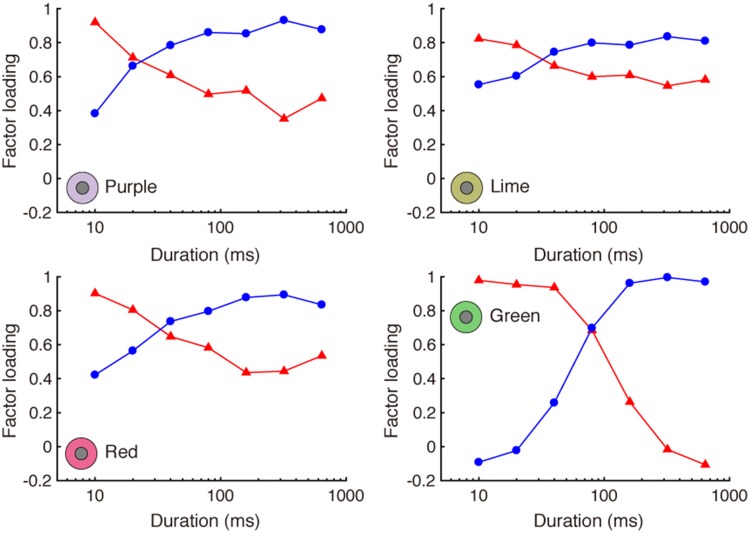
Factor loading for two factors, for each color inducer condition. One factor has high loading at short (≤40 ms) durations, and the other has high loading at longer (>80 ms).

We applied factor analysis to each of the four inducer conditions separately. For all four conditions, a two-factor solution was found to be optimal, and factor loadings were tuned systematically across duration. Factor loadings for each inducer condition are shown in Figure 7. Again, the two factors were clearly different depending on stimulus duration. One factor (fast) has high loadings at shorter durations, and the other factor (slow) has high loadings at longer durations. The two factors accounted for 98.5% (purple), 99.1% (lime), 97.6% (red), and 96.0% (green) of the total variance.

As predicted, the factors were consistent with fast and slow processes operating over short and long presentation durations. Although the fast and slow factor loading patterns differed slightly across conditions, with the two factors for the green surround being most distinct, they emerged reliably for the four chromatic surround conditions.

## Color Experiment 2

The last data set we reanalyzed was taken from a single experiment reported by Kaneko et al. (2017, Experiment 2 of 2) and listed as Color Experiment 2 in Table 1.

Kaneko et al. measured induction strength for conditions in which the test, in addition to the inducer, was chromatic. A central test stimulus was first set to unique yellow (appearing neither reddish nor greenish). On each trial, it was viewed within one of four concentric surrounds ranging in chromaticity (red, reddish yellow, greenish yellow, and green, varying in hue but not saturation). In this experiment, Kaneko et al. (2017) examined the effect of stimulus duration on simultaneous color contrast by comparing induction effects for 10 ms and 500 ms.

### Methods

#### Observers

Five observers with normal or corrected-to-normal visual acuity participated. Their age was between 18 and 28 years.

#### Apparatus and stimuli

Stimuli were presented on a 19″ CRT display (Sony CPD-G400, refresh rate 100 Hz) under computer control. All stimuli were generated using the MATLAB programming environment with Psychtoolbox (Brainard, 1997; Pelli, 1997). The test stimulus was a 1° diameter disk with a thin black contour and was embedded at the center of a uniformly colored 8° diameter inducing disk. All the colors in this experiment were chosen from an equiluminant cone-opponent color space, whose origin was the equal-energy white (20 cd/m^2^). Amplitude of the L-M modulation was 7% in L-cone contrast, and the amplitude of the S modulation was 70% in S-cone contrast of the origin, equal-energy white. Heterochromatic flicker photometry was performed for each observer to ensure equiluminance of the colors. Hues were defined by azimuth in this color space with 0° being +L-M and 90° being +S. The hue of the inducer was chosen relative to each observer’s unique yellow (average azimuth of unique yellow was 294°; note that henceforth 0° indicates this individual unique yellow hue). Inducer hue conditions were −60° (“green”), −12° (“greenish yellow”), 0°, +12° (“reddish yellow”), and +60° (“red”), where the positive value indicates a counter-clockwise hue shift from the unique yellow (the inducer hue 0° condition was a control condition and excluded from the later factor analysis). Each observer’s unique yellow azimuth was measured separately before Color Experiment 2. The duration of the test stimulus was either 10 ms or 500 ms. Different duration conditions were run in separate sessions.

#### Procedure

Unlike the other experiments from Kaneko and Murakami (2012), cancellation method was employed in Color Experiment 2. The test and the inducer were presented at the center of the display for 10 ms or 500 ms. Observers viewed the stimuli foveally and indicated by a keypress whether the hue of the test was closer to “red” or “green.” If the observer’s response was “red,” the test hue was shifted in the greener (negative) direction. No instruction was given as to the definitions of “red” and “green.” The hue of the test was changed according to the staircase method (final step size was 3.75°). The average azimuth of the last four reversal points of the staircase was taken as the new unique yellow (unique yellow measured with a colored inducer) for that condition. The azimuth difference between the original unique yellow (unique yellow measured before Color Experiment 2) and the new unique yellow was calculated as the hue shift.

The experimental procedure was approved by the institutional review board of The University of California, San Diego, where all the experiments were performed. All observers gave their written informed consent.

### Results: Group Averages (Kaneko et al., 2017)

The red and reddish yellow surrounds induced perception of the yellow test to appear more greenish, while the greenish yellow and green surrounds induced perception of the test to appear more reddish. For all four conditions, consistent with Kaneko and Murakami (2012), the perceptual shifts away from unique yellow were significantly greater for 10 ms than 500 ms presentations.

**Figure 8.**
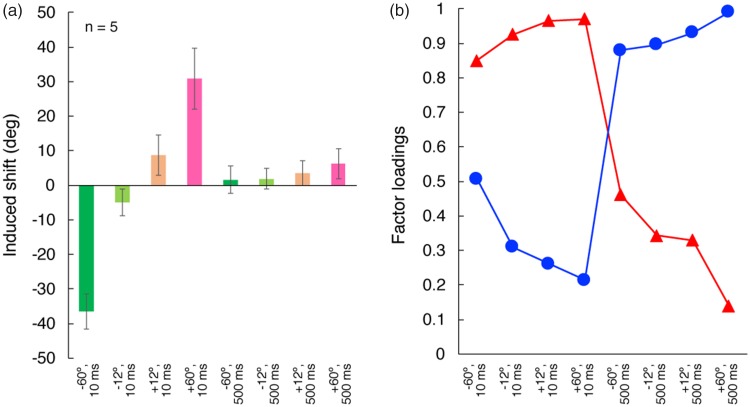
(a) Induced shift of “unique yellow” with different inducers. Mean of five observers’ data are shown. Error bars indicate standard errors. (b) Factor loading for two factors. One factor had high loadings at 10 ms but low loadings at 500 ms, while the other factor had the opposite loading pattern.

The data supporting these conclusions are shown in Figure 8(a). The mean-induced shift (in azimuth) for five observers is shown. The azimuth of the new unique yellow minus the azimuth of the original unique yellow represented the induced shift in this experiment. Because the cancellation method was used, a significant shift of the same sign as that of the inducer would be found whenever simultaneous contrast occurred. The amount of this induced hue shift was taken as the measure of induction strength. Figure 8(a) clearly shows that the induced hue shift was significantly stronger at 10 ms than at 500 ms.

### Results: Factor Analysis of Kaneko et al. (2017) Data

#### Predictions

Again, for this data set, we predicted that individual variability in the data would reveal factors from the individual differences consistent with fast and slow processes operating over different duration ranges (short and long, respectively, consistent with Kaneko & Murakami, 2012). Separate fast and slow processes were predicted to emerge reliably for the 10 ms and 500 ms conditions, respectively, with one factor accounting for the four surround conditions at 10 ms, and another accounting for the four surround conditions at 500 ms.

#### Results and discussion

For this data set, the initial PCA found a two-factor solution to be optimal. Factor loadings for two Varimax-rotated factors are shown in Figure 8(b). The plot shows two clearly defined factors. One factor (fast) has high loadings only at 10 ms, regardless of the inducer hue conditions, and the other factor (slow) has high loadings only at 500 ms. The two factors accounted for 97.5% of the total variance. Thus, the individual variability in these data reveal factors that are consistent with fast and slow processes operating over different duration ranges (short and long, respectively).

## General Discussion

In the current study, we tested a theory proposed by Kaneko and Murakami (2012) regarding the mechanisms underlying simultaneous contrast effect. The theory postulates that there are fast and slow processes involved in the simultaneous contrast effect. The fast process dominates the effect when the stimulus duration is very short and yields a strong effect, whereas the slow process becomes dominant only when the stimulus duration is sufficiently long and yields a weak effect (Kaneko & Murakami, 2012).

To test this theory, we revisited archival data sets from Kaneko and Murakami (2012), as well as an additional archival data set from Kaneko et al. (2017), and performed factor analyses on them. After completing 12 different factor analyses obtained from 12 different sets of data obtained from these archival studies, and examining the factor loadings, we consistently obtained separate factors tuned to short and long durations. Considering that sample size for each analysis was only five to seven observers, we think it is remarkable to find such clear and reliable results.

The outcome of each of our 12 factor analyses is consistent with what Kaneko and Murakami (2012) hypothesized. There is one process (factor) that transiently responds to the stimulus onset and produces strong simultaneous contrast and another process that responds with some delay to yield weaker simultaneous contrast. Kaneko and Murakami (2012) also predicted that the duration where the slow process becomes more dominant than the fast process would be 49 ms (mean of three observers who participated in both Brightness Experiment 2 and Color Experiment 1), which is also quantitatively similar to what we found for two factor solutions and the factor loading curves for different durations (see Figures 5 and 7).

The factor analyses not only confirmed the hypothesis and model of Kaneko and Murakami (2012) but provided a preliminary exploration of the processes underlying separate features. That is, the factor analyses suggest a possible separation of processes for different polarities. Specifically, Brightness Experiment 1 clearly showed that the increments (inducer being lighter than the test) and decrements (inducer being dimmer than the test) are processed separately, that is, by different factors. There are previous studies that reported asymmetry in the simultaneous brightness contrast between increment and decrement, that is, a larger effect when the inducer was lighter than the test (for a classical example, see Heinemann, 1955). Also, it is widely held that the luminance increment and decrement are processed separately via on- and off-channels, which originate in the retina (e.g., Schiller, 1992). Therefore, it is not surprising to see such separate factors for different luminance polarities. However, it is noteworthy that this did not emerge from simply observing the mean results from Kaneko and Murakami (2012), since there was little indication of these separate factors either quantitatively or qualitatively. From the current factor analysis, we conclude tentatively, based on these small sample analyses, that there are fast and slow processes involved in the simultaneous brightness contrast, but also that the two processes for increment conditions are separate from the two processes for decrement conditions.

From Brightness Experiment 2, we found a fast factor that has high loadings at short duration conditions and a slow factor that has high loadings at long duration conditions for each inducer condition. However, the distinction between the fast and slow factors were less clear for decrement inducers than for increment inducers, with double crossovers in loadings (see Figure 5). We speculate that this difference may suggest slightly different temporal tunings of fast and slow mechanisms for decrements and increments, though this outcome could also reflect random effects caused by small sample size. In fact, Gunther and Dobkins (2005) examined the brightness induction effect with flickering stimuli and reported that the brightness induction effect was strong at low (4 Hz) and high (>20 Hz) temporal frequencies while the effect is weak at mid-range temporal frequencies, in other words, double crossover (see Gunther & Dobkins, 2005; Figure 2). In addition, it has been suggested that the processing of luminance increment and decrement is separate and temporally different (e.g., Wehrhahn & Rapf, 1992). Taken together, the seemingly mysterious loading profiles of fast and slow factors seen for decrement inducers in Brightness Experiment 2 could possibly indicate the true nature of the underlying mechanisms.

From Color Experiment 1, we also found satisfactory two-factor solutions for each inducer condition. At durations ranging from 20 to 80 ms, there was a transition from one factor having a higher loading to another having higher loading. This duration range where the switch between factors occurred was also similar to the pattern observed in the Brightness Experiment 2 (see Figure 5). Kaneko and Murakami (2012) did not emphasize the separation between fast and slow processes for color contrast. This was because the spatial gap between the test and the inducer affected the color induction strength similarly for 10 ms and 500 ms conditions (Kaneko & Murakami [2012], Experiment 3, not included in the current analyses), whereas the same spatial gap reduced 10-ms simultaneous brightness contrast but not 500 ms. Similar spatial properties between 10 ms and 500 ms effects for simultaneous color contrast suggested by this finding refrained Kaneko and Murakami (2012) from proposing two separate fast and slow processes. That we nonetheless observed clear fast and slow factors from the color data probably suggest that Kaneko and Murakami (2012)’s original color experiment was not sensitive enough to differentiate fast and slow processes, or these mechanisms are perhaps not different spatially.

The factor analysis for Color Experiment 2, for data originally collected by Kaneko et al. (2017), also found two-factor solutions, where each factor had high loadings for either 10 ms conditions or 500 ms conditions. This experiment was conducted in a different laboratory with observers who did not participate previously in any of the experiments of Kaneko and Murakami (2012). Nonetheless, fast and slow factors were found in this data set despite such differences, further supporting the hypothesis of Kaneko and Murakami (2012).

We believe that the current study reliably supports the theory of Kaneko and Murakami (2012) that there are separate fast and slow temporal processes underlying simultaneous contrast for brightness and color. The study demonstrated that reanalyzing the archival data using factor analyses of individual variability provided converging evidence to support the theory. Moreover, it demonstrated that this converging evidence can sometimes be obtained reliably across studies even for very small samples. To be sure, adding more participants to samples like these will lead to more clarity about underlying processes. Quick snapshots can provide a blurry image, but a blurry snapshot can still provide some unequivocal and new details about a scene. Similarly, our small sample analyses, while perhaps “blurry,” provide unequivocal and new results which strongly confirm the two-process model of simultaneous color and brightness contrast (Kaneko & Murakami, 2012).

## Note

1 In this article, we use the terms “luminance” to refer to the physical measure of light intensity and “brightness” to refer to the perceived light intensity.
